# The rs878081 polymorphism of *AIRE* gene increases the risk of rheumatoid arthritis in a Chinese Han population: a case-control study

**DOI:** 10.1590/1414-431X20187944

**Published:** 2018-11-01

**Authors:** Haoyu Yang, Jin Li, Lifeng Jiang, Xijia Jiang, Xindie Zhou, Nanwei Xu

**Affiliations:** 1 Department of Orthopedics, The Affiliated Changzhou No.2 People's Hospital of Nanjing Medical University, Changzhou, Jiangsu Province, China; 2 Department of Orthopedic Surgery, The Second Affiliated Hospital, Zhejiang University School of Medicine, Hangzhou, China; 3 Department of Orthopedic Surgery, The Second Affiliated Hospital of Jiaxing University, Jiaxing City, Zhejiang Province, China

**Keywords:** AIRE, Single-nucleotide polymorphism, Rheumatoid arthritis, Case-control study

## Abstract

The autoimmune regulator (AIRE), a transcriptional regulator expressed in medullary thymic epithelial cells, plays an important role in thymocyte education and negative selection. Several citations studying the association between the rs878081 exon polymorphism of the *AIRE* gene and the risk of rheumatoid arthritis (RA) in different populations have yielded conflicting findings. Thus, this case-control study involving 300 RA cases and 300 controls was aimed to identify whether such association existed in a Chinese Han population from East China. The rs878081 polymorphism of the *AIRE* gene was genotyped. Odds ratios (ORs) and 95% confidence intervals (CIs) were estimated using the chi-squared test, genetic model analysis, and stratification analysis. Genetic model analysis showed significant correlations between the TT genotype and the risk of RA (OR: 1.89, 95%CI: 1.03-3.47 in TT *vs* CC; OR: 1.84, 95%CI: 1.02-3.31 in TT *vs* CC+TC). Stratification analyses of sex, age, smoking, and alcoholism suggested that the rs878081 polymorphism of the *AIRE* gene increased RA risk among non-smokers. In conclusion, rs878081 polymorphism of *AIRE* gene increases the risk of RA in a Chinese Han population.

## Introduction

Rheumatoid arthritis (RA), an autoimmune disease affected by multiple factors (1), could cause critical functional impairment and work-related disability despite its low prevalence (∼1%) (1). No significant reduction in mortality in different RA populations worldwide has been demonstrated (2,3). It has also been reported that the improvement in survival lags behind the recent advances in RA management (2). In addition, the etiology and pathogenesis of RA are still unknown (4). The combination of environmental and genetic factors plays a major regulating role in the development and progression of RA.

The autoimmune regulator (AIRE) is a transcriptional regulator expressed in thymic medullary cells (5). Experimental studies show that single nucleotide polymorphisms (SNPs) alter transcription of the *AIRE* gene, which is located in the 21q22.3 region, ∼12.5 kb long, encoding a 545 amino acid protein of 58 kDa by 14 exonial sequences (6). SNPs thereby provide a less efficient negative selection, and improve the survival of autoimmune T-cells and the susceptibility to autoimmune diseases (7–9). A genome-wide association study in a Japanese population identified two SNPs (rs2075876 and rs760426) in the *AIRE* gene significantly associated with RA risk (10). Moreover, several citations (11-13) studying the association between the *AIRE* gene rs878081 polymorphism and RA risk in different populations brought conflicting findings. According to dbSNP database, rs878081 is located in the exon region of the *AIRE* gene. The minor allele frequency (MAF) of T allele in rs878081 polymorphism was 0.133 according to the 1000 Genomes Browser (https://www.ncbi.nlm.nih.gov/variation/tools/1000genomes/). The MAFs of T allele in rs878081 polymorphism in the cases were 0.196, 0.074, and 0.290 in a Spanish population (11), a Chinese population from Hebei province (12), and a Chinese population from Shanxi province (13), respectively. To date, there is no association study of this SNP among the Chinese Han population in East China. Thus, this case-control study aimed to investigate whether rs878081 polymorphism of the *AIRE* gene was associated with the risk and development of RA in a Chinese Han population from Jiangsu province (East China).

## Material and Methods

### Patients and methods

In this hospital-based case-control design, 300 hospitalized RA patients (79 males and 221 females) were recruited from the Affiliated Changzhou No.2 People's Hospital of Nanjing Medical University or the Second Affiliated Hospital of Jiaxing University between January 2014 and October 2017. They were diagnosed following the criteria of the American College of Rheumatology/European League against Rheumatism Collaborative Initiative for RA (14). All patients were clinically evaluated by two rheumatologists. The demographic and RA data were obtained from patient data sheets. All the recruited people were Chinese Han.

Patients with other nationalities, consanguinity, other major systemic disease, collagen vascular disease, other autoimmune diseases, hepatitis C virus infection, or family history of autoimmune diseases were excluded. Also, 300 unrelated healthy controls (128 males and 172 females) were randomly recruited from the subjects receiving regular health examinations at either of the two hospitals during the same period.

RA activity was measured using the Disease Activity Score in 28 joints and the erythrocyte sedimentation rate (DAS28-ESR) (15), which evaluates the tenderness and swelling in 28 joints. Grades of disease activity were classified as follows: high ≥5.1, moderate <5.1 and ≥3.2, low <3.2 and ≥2.6, and clinical remission <2.6.

Both serum and ethylene-diamine-tetra-acetic acid (EDTA) blood samples were collected from both groups for measurement of rheumatoid factor (RF), C-reactive protein (CRP), erythrocyte sedimentation rate (ESR), anti-cyclic citrullinated peptide (anti-CCP) antibodies, and *AIRE* gene polymorphism. Both RF and CRP were measured using a nephelometer (Turbox plus analyzer, Orion Diagnostica, Finland). Anti-CCP antibodies were analyzed by third-generation enzyme-linked immunosorbent assay (ELISA; CCP3 IgG, Inova Quanta Lite^TM^, USA). The cut-off value for CRP was ≥10 mg/L, for RF was ≥25 IU/mL, and for anti-CCP antibodies was <20 negative, 20-39 weakly positive, 40-59 moderately positive, and ≥60 strongly positive.

A detailed questionnaire about smoking and alcoholism habits was completed for each participant by a trained interviewer. Informed consent was obtained from all participants prior to their participation. Ethical approval, which was in line with the Declaration of Helsinki standards, for the study protocol was obtained from the Ethics Committees of the two Hospitals.

### DNA extraction and genotyping

Blood samples were collected using vacutainer tubes and then transferred to EDTA tubes. Genomic DNA was isolated from whole blood using a QIAamp DNA blood mini kit (Qiagen, Germany). Genotyping was performed by matrix-assisted laser desorption/ionization time-of-flight mass spectrometry (MALDI-TOF MS) as previously described (16). SNP was genotyped by the MassARRAY system (Sequenom, USA) and by MALDI-TOF MS, which was performed without knowing patient status (case *vs* control) to ensure the quality of genotyping.

### Genotype and gene expression correlation analysis

Genotype data of the *AIRE* gene rs878081 polymorphism were available online from the International HapMap Project. The mRNA expression data were available online from Genotype-Tissue Expression Portal (https://www.gtexportal.org/home/) (17).

### mRNA extraction and real-time PCR

According to the manufacturer's protocol, mRNAs were extracted from blood samples using TRIzol reagent (Invitrogen; Thermo Fisher Scientific, Inc, USA). cDNA was synthesized using a PrimeScript RT Reagent kit (Takara Biotechnology Co., Ltd., China) at 37°C for 15 min, 85°C for 5 s, and determined at 4°C, while qPCR was conducted using a SYBR Premix Ex Taq kit (Takara Biotechnology Co., Ltd.) on a Bio-Rad iQ5 Real-Time PCR system (Bio-Rad Laboratories, Inc., USA). The following thermocycling conditions were used for the PCR: 50°C for 2 min, 95°C for 10 min, followed by 40 cycles of 95°C for 15 s and 60°C for 1 min. The primer sequences for amplification were 5′-GAGAGTGCTGAGAAGGACA-3′ (forward) and 5′-GTTTAATTTCCAGGCACATGA-3′ (reverse). The relative expression was calculated using the 2^-ΔΔCq^ method, with glyceraldehyde-3-phosphate dehydrogenase (GAPDH) used as the internal control.

### Statistical analysis

Demographic characteristics and rs878081 genotypes of the *AIRE* gene were evaluated using a chi-squared test (χ^2^) (for categorical variables) or Student's *t*-test (for continuous variables). The associations between the rs878081 T/C genotypes and the risk of RA were estimated by calculating odds ratios (ORs) and 95% confidence intervals (CIs) using logistic regression analysis; crude ORs and adjusted ORs were used in case of adjustment for age and sex. The Hardy-Weinberg equilibrium (HWE) was assessed by a goodness-of-fit χ^2^ test to compare the observed genotype frequencies with the expected frequencies in controls. This study was powered to detect the effect of rs878081 polymorphism of the *AIRE* gene on RA susceptibility at P<0.05 (18). All statistical analyses were performed on SAS software 9.1.3 (SAS Institute, USA).

## Results

### Characteristics of the study population

The characteristics of the study population are summarized in Table 1, including DAS28 grades and morning stiffness. The case and control groups were well-matched in terms of gender (male proportion=44.67% *vs* 48.67%) and age (mean age=54.50 *vs* 53.84 years). No significant differences between groups were found in smoking or alcoholism.

**Table 1.** Demographics and baseline characteristics of rheumatoid arthritis patients.

	Patient (n = 300)	Control (n = 300)
Age, years (mean±SD)	54.5 ± 15.48	53.84 ± 10.48
Gender (male / female)	134 / 166	146 / 154
Smoking (yes / no)	120 / 180	121 / 179
Alcohol (yes / no)	87 / 213	86 / 214
Disease duration, years (mean±SD)	9.10 ± 9.35	
Disease onset (age; mean±SD)	45.46 ± 12.58	
Family history (yes / no)	76 / 224	
DAS28 (median ± SD)	4.35 ± 1.61	
DAS28 grade (n, %)		
High activity	25 (8.3%)	
Moderate	110 (36.7%)	
Low	132 (44.0%)	
Remission	33 (11.0%)	
N of tender joints (mean±SD)	8.68 ± 5.84	
N of swollen joints (mean±SD)	10.26 ± 5.53	
Deformity (n)	113 / 187	
Morning stiffness (n, %)		
None	60 (20.0%)	
≤1.0 h	169 (56.3%)	
>1.0 h	71 (23.7%)	
Positive / Negative Rheumatoid factor (n)	241 / 59	
Positive / Negative CRP (n)	185 / 115	
ESR (median ± SD)	33.84 ± 21.85	
Positive / Negative Anti-CCP (n)	165 / 135	

DAS28: disease activity score in 28 joints; CRP: C-reactive protein; ESR: erythrocyte sedimentation rate; CCP: cyclic citrullinated peptide.

### Association between rs878081 polymorphism and RA risk

Genotype distributions of the rs878081 polymorphism of the *AIRE* gene among all subjects are reported in Table 2. The rate of genotyping was >98%, and the test polymorphism of the study population was under HWE (P>0.05). Logistic regression analyses revealed the rs878081 polymorphism increased the risk of RA in two genetic models (OR: 1.89, 95%CI: 1.03-3.47 in TT *vs* CC, P=0.039 and OR: 1.84, 95%CI: 1.02-3.31 in TT *vs* CC+TC, P=0.043; Table 2). The effects of the SNP on RA risk were further evaluated according to age, gender, smoking, and drinking. The increased RA risk conferred by rs878081 was more significant in females and non-smoking patients (Table 3). However, no significant association was found between rs878081 genotypes and clinical or biochemical characteristics except for tender joints (P=0.020, Table 4). Furthermore, no significant difference in demographic or laboratory data was found between CC+CT and TT genotypes (Table 5) or between CC and CT+TT genotypes (Table 6).

**Table 2.** Distribution of autoimmune regulator (AIRE) gene rs878081 alleles and genotypes in rheumatoid arthritis patients and healthy controls.

AIRE gene polymorphism	Patients (n = 298) (n, %)	Control (n = 299) (n, %)	OR (95%CI)	P value
Allele				
C	421 (70.6%)	450 (75.3%)	Reference	
T	175 (29.4%)	148 (24.7%)	1.26; (0.98-1.63)	0.073
Genotype				
CC	156 (52.3%)	170 (56.9%)	Reference	
CT	109 (36.6%)	110 (36.8%)	1.08; (0.77-1.52)	0.660
TT	33 (11.1%)	19 (6.3%)	1.89; (1.03-3.47)	0.039
CC+CT *vs* TT	265 / 33 (88.9 / 11.1)	280 / 19 (93.6 / 6.4)	1.84; (1.02-3.31)	0.043
TT+CT *vs* CC	142 / 156 (47.7 / 52.3)	129 / 170 (43.1 / 56.9)	1.19; (0.86-1.66)	0.269
HWE		0.831		

HWE: Hardy Weinberg equation.

**Table 3.** Stratified analyses between *rs878081* polymorphism and the risk of rheumatoid arthritis stratified by factors.

Variable	*Rs878081* (case/control)			OR (95%CI); P			
	CC	CT	TT	CT *vs* CC	TT *vs* CC	CT+TT *vs* CC	TT *vs* CT+CC
Sex							
Male	73/88	51/51	10/7	1.21	1.72	1.27	1.60
				(0.73–1.98)	(0.62–4.75)	(0.79–2.04)	(0.59–4.34)
				0.461	0.294	0.327	0.354
Female	83/82	58/59	23/12	0.96	1.87	1.13	1.92
				(0.60–1.54)	(0.88–4.00)	(0.73–1.75)	(0.92–4.00)
				0.865	0.106	0.595	0.083
Age							
≥55	80/84	61/44	15/6	1.44	2.59	0.95	2.27
				(0.88–2.36)	(0.96–7.01)	(0.60–1.48)	(0.85–6.02)
				0.150	0.061	0.807	0.100
<55	76/86	48/66	18/13	0.82	1.56	1.60	1.70
				(0.51–1.33)	(0.72–3.40)	(1.00–2.55)	(0.80–3.60)
				0.425	0.259	0.051	0.168
Smoking							
Yes	77/69	35/45	7/7	0.69	0.89	0.72	1.02
				(0.40–1.19)	(0.30–2.65)	(0.43–1.22)	(0.35–3.00)
				0.181	0.827	0.224	0.974
No	79/101	74/65	26/12	**1.45**	**2.76**	**1.66**	**2.35**
				**(0.93–2.26)**	**(1.31–5.81)**	**(1.09–2.52)**	**(1.15–4.82)**
				**0.010**	**0.007**	**0.018**	**0.020**
Alcoholism							
Yes	50/53	30/30	7/3	1.06	2.47	1.19	2.42
				(0.56–2.00)	(0.61–10.09)	(0.65–2.18)	(0.61–9.68)
				0.858	0.207	0.578	0.212
No	106/117	79/80	26/16	1.08	1.78	1.21	1.73
				(0.72–1.62)	(0.90–3.49)	(0.82–1.77)	(0.90–3.33)
				0.714	0.096	0.334	0.101

Bold type indicates statistically significant.

**Table 4.** Comparison of studied data according to autoimmune regulator (AIRE) gene genotypes in all rheumatoid arthritis (RA) cases.

	RA			P
	CC (n = 156)	CT (n = 109)	TT (n = 33)	
Age (years; mean±SD)	54.48±15.95	54.93±15.48	53.45±13.42	0.891
Males (n, %)	73 (46.8%)	51 (46.8%)	10 (30.3%)	0.199
Females (n, %)	83 (53.2%)	58 (53.2%)	23 (69.7%)	
Family history (n, %)	39 (25.0%)	32 (29.4%)	5 (15.2%)	0.255
Onset (years; median±SD)	45.47±12.72	45.91±13.11	43.91±10.16	0.728
Morning stiffness (n, %)				0.557
0	29 (18.6%)	24 (22.0%)	7 (21.2%)	
<1 hour	93 (59.6%)	59 (54.1%)	15 (45.5%)	
>1 hour	34 (21.8%)	26 (23.9%)	11 (33.3%)	
Deformities (n, %)	57 (36.5%)	46 (42.2%)	9 (27.3%)	0.278
ESR (median±SD)	34.15±21.92	32.70±21.52	36.82±23.20	0.628
Positive CRP (n, %)	91 (58.3%)	70 (64.2%)	22 (66.7%)	0.504
Positive RF (n, %)	127 (81.4%)	86 (78.9%)	27 (81.8%)	0.862
Positive anti-CCP (n, %)	88 (56.45%)	56 (51.4%)	21 (63.6%)	0.431
Tender joints (median±SD)	9.22±5.99	7.48±5.49	10.06±5.83	0.020
Swollen joints (median±SD)	10.42±5.66	10.19±5.52	9.58±5.15	0.727
DAS (median±SD)	4.33±1.57	4.39±1.70	4.32±1.53	0.946

ESR: erythrocyte sedimentation rate; CRP: C-reactive protein; RF: rheumatoid factor; CCP: cyclic citrullinated peptide; DAS: disease activity score in 28 joints. Statistical analyses were carried out with one-way ANOVA and the chi-squared test.

**Table 5.** Comparison of studied data according to autoimmune regulator (AIRE) genotypes in all rheumatoid arthritis (RA) cases.

	RA (n = 300)		P
	CC + CT (n = 265)	TT (n = 33)	
Age (years, mean±SD)	54.66±15.73	53.45±13.42	0.673
Males (n, %)	124 (46.8%)	10 (30.3%)	0.073
Females (n, %)	141 (53.2%)	23 (69.7%)	
Family history (n, %)	71 (26.8%)	5 (15.2%)	0.148
Onset (years, median±SD)	45.65±12.86	43.91±10.16	0.455
Morning stiffness (n, %)			0.338
0	53 (20.0%)	7 (21.2%)	
<1 hour	152 (57.4%)	15 (45.5%)	
>1 hour	60 (22.6%)	11 (33.3%)	
Deformities (n, %)	103 (38.9%)	9 (27.3%)	0.195
ESR (median±SD)	33.55±21.73	36.82±23.20	0.419
Positive CRP (n, %)	161 (60.8%)	22 (66.7%)	0.511
Positive RF (n, %)	213 (80.4%)	27 (81.8%)	0.844
Tender joints (median±SD)	8.51±5.84	10.06±5.83	0.150
Swollen joints (median±SD)	10.32±5.59	9.58±5.15	0.465
DAS (median±SD)	4.36±1.62	4.32±1.53	0.905

ESR: erythrocyte sedimentation rate; CRP: C-reactive protein; RF: rheumatoid factor; DAS: disease activity score in 28 joints. Statistical analyses were carried out with the *t*-test and chi-squared test.

**Table 6.** Comparison of studied data according to autoimmune regulator (AIRE) genotypes in all rheumatoid arthritis (RA) cases.

	RA (n = 300)		P
	CC (n = 156)	CT+TT (n = 142)	
Age (years, mean±SD)	54.48±15.95	54.58±15.00	0.954
Males (n, %)	73 (46.8%)	61 (43.0%)	0.506
Females (n, %)	83 (53.2%)	81 (57.0%)	
Family history (n, %)	39 (25.0%)	37 (26.1%)	0.834
Onset (years, median±SD)	45.47±12.72	45.44±12.48	0.987
Morning stiffness (n, %)			0.351
0	29 (18.6%)	31 (21.8%)	
<1 hour	93 (59.6%)	74 (52.1%)	
>1 hour	34 (21.8%)	37 (26.1%)	
Deformities (n, %)	57 (36.5%)	55 (38.7%)	0.696
ESR (median±SD)	34.15±21.92	33.65±21.90	0.846
Positive CRP (n, %)	91 (58.3%)	92 (64.8%)	0.253
Positive RF (n, %)	127 (81.4%)	113 (79.6%)	0.690
Tender joints (median±SD)	9.22±5.99	8.08±5.66	0.091
Swollen joints (median±SD)	10.42±5.66	10.05±5.42	0.568
DAS (median±SD)	4.33±1.57	4.38±1.66	0.808

RF: rheumatoid factor; CRP: C-reactive protein; DAS: disease activity score in 28 joints. Statistical analyses were carried out with the *t*-test and chi-squared test.

### Association between rs878081 polymorphism and RA population

**Figure 1.**
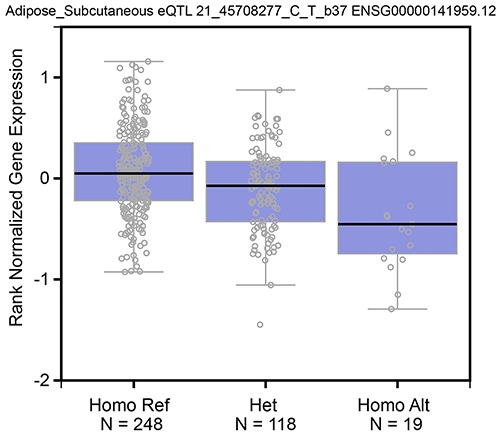
Proteins interacting with autoimmune regulator (AIRE). Homo ref: CC; Het: CT; Homo Alt: TT.

According to dbSNP database and Genotype-Tissue Expression Portal (17), the TT genotype decreased the AIRE mRNA levels compared to CC genotype in the adipose-subcutaneous (Figure 1). A significant difference was found in the expression levels for rs878081 polymorphism with the expression quantitative trait loci (eQTL) analysis (P<0.01).

**Figure 2.**
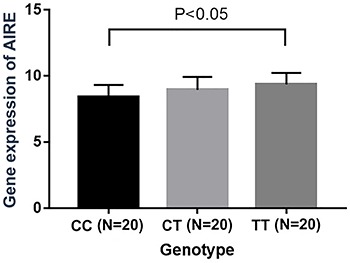
Expression levels of autoimmune regulator (AIRE). Data are reported as arbitrary units. P<0.05 (*t*-test).

Similar results were observed in the patients' blood samples. A statistically significant difference in the mean levels of expression of the rs878081 alleles was found (P=0.018). The transcription of AIRE was decreased by the T allele compared with the C allele (Figure 2).

## Discussion

The association between the rs878081 polymorphism of the *AIRE* gene and the risk of RA was investigated in a Chinese population, which showed that the polymorphism conferred susceptibility to RA.

AIRE plays a functional role in thymocyte education and negative selection by controlling the thymus expression of peripheral antigens (5,19). Dysfunction of the *AIRE* gene could lead to autoimmune polyendocrinopathy-candidiasis-ectodermal dystrophy (20,21). The association between several SNPs (rs2075876, rs760426, rs1800250, rs2776377, rs1055311, rs933150, rs1003854, rs2256817, rs374696, rs1078480) of the AIRE gene and RA risk was studied before, and a recent meta-analysis (22) proved that rs2075876 and rs760426 are significantly associated with an increased risk of RA. After genotype-tissue expression analysis indicated that TT genotype of rs878081 polymorphism significantly decreased the AIRE expression, many researchers focused on the association between *AIRE* rs878081 polymorphism and RA risk. Garcia et al. (11) showed that the minor allele of rs878081 was significantly more frequent in RA patients than in controls from Spain, which was in line with our findings. However, two studies (12,13) from West and North China, showed no evidence of association between rs878081 and RA risk. Such discrepancies could be attributed to four reasons. First, genetic heterogeneity may exist among populations and living environments, but RA results from the intricate interactions between various susceptibility genes and environmental factors. The effects of some genetic variants may vary across different populations and environments (1). Second, the study designs were different. The Spanish study (11) included fewer females in the RA group than the two Chinese studies (12,13). Moreover, cases in the West China study (13) were considerably younger than cases in the North China study (mean age=43.5 *vs* 53.9 years). Genetic factors are often stronger in younger cases, and menopause status is an influential factor (23). Third, clinical heterogeneity may exist among studies. Fourth, since the sample sizes of some studies were too small to draw a convincing conclusion, the results may be false-positives or false-negatives. Nevertheless, a meta-analysis including this SNP is warranted to resolve inconsistence and evaluate the role of this SNP in the risk of RA.

Stratification analyses showed that the risk of RA conferred by the rs878081 polymorphism of the *AIRE* gene remained significant in the non-smoking subgroup, which was because susceptible individuals were likely to be exposed to risk factors to some extent. In addition, the genotype distribution of rs878081 polymorphism in relation to demographic, clinical, and laboratory data was analyzed. Individuals with TT genotype had more tender joints. However, given the decreased sample sizes in the stratification analyses and the limited power, the results should be interpreted with caution. Nevertheless, our findings still provide evidence for a possible interaction between the SNP and some RA risk factors.

Our results indicated that the *AIRE* rs878081 polymorphism increased the risk of RA in a Chinese population. According to dbSNP database, rs878081 polymorphism will cause synonymous mutation when the nucleotide changes from C to T. Then, we investigated the mRNA levels of different genotypes of rs878081 polymorphism in the Genotype-Tissue Expression Portal (17). The TT genotype decreased the AIRE mRNA levels compare to CC genotype in the adipose-subcutaneous. Considering that our clinical specimen was whole blood, we extracted mRNA from blood sample and conducted qPCR to determine the levels of AIRE. Similar results were observed in the patients' serum. Above all, the proportion of TT genotype increased in the RA group compared to the normal group. TT genotype is more likely to cause the down-regulation of AIRE compared to CC or CT genotypes. We hypothesized that AIRE rs878081 polymorphism conferred susceptibility to RA by altering the expression levels of AIRE.

The association between the *AIRE* rs878081 polymorphism and risk of other autoimmune diseases was examined before (6,24–26). Ferrera et al. (24) reported no significant association of this SNP with risk of systemic sclerosis in an Italian population. No significant association was found between rs878081 polymorphism and Grave's disease (6), Addison's disease (26), or type 1 diabetes (25). However, some studies demonstrated that the ARIE rs878081 polymorphism conferred susceptibility to RA. The above differences among studies may be explained by the disease-dependent functionality of rs878081 polymorphism and should be confirmed by further studies.

This case-control study has several potential limitations that merit careful consideration. First, the patients and controls recruited from hospitals may not be representative of the general population. Nonetheless, the genotype distribution of the controls was in HWE. Second, because of the limited sample size, a single case-control study may be insufficient to fully uncover the relationship between the *AIRE* rs878081 polymorphism and susceptibility to RA. Thus, our findings should be confirmed by larger numbers of subjects. Third, we did not obtain detailed information about RA severity and response to treatment, which restricted our analyses. Fourth, the risk of RA cannot be attributed to a single *AIRE* gene SNP, but should be interpreted by considering other *AIRE* SNPs, other genes, and some environmental factors. Fifth, the underlying mechanisms of this SNP in RA were not investigated. Finally, the true significance of the association between this SNP and RA risk should be validated by further studies in different populations.

In conclusion, our study provided strong evidence that the rs878081 polymorphism of the *AIRE* gene may contribute to RA risk. However, this finding was obtained with a limited sample size representing a preliminary conclusion and should be confirmed by multicenter case-control studies with diverse ethnic populations.
